# Increasing women’s access to skilled pregnancy care to reduce maternal and perinatal mortality in rural Edo State, Nigeria: a randomized controlled trial

**DOI:** 10.1186/s41256-018-0066-y

**Published:** 2018-04-04

**Authors:** Sanni Yaya, Friday Okonofua, Lorretta Ntoimo, Bernard Kadio, Rodrigue Deuboue, Wilson Imongan, Wapada Balami

**Affiliations:** 1 School of International Development and Global Studies, University of Ottawa, Ottawa, Canada; 2 Women’s Health and Action Research Centre, Benin City, Nigeria; 3 Federal University Oye-Ekiti, Oye-Ekiti, Ekiti State Nigeria; 4 Hospital Service Department, Federal Ministry of Health, Abuja, Nigeria

**Keywords:** Maternal health, Newborn health, Health policy, Global health, Randomized Controlled Trial, Nigeria, Maternal and perinatal mortality

## Abstract

**Background:**

Nigeria presently has the second highest absolute number of maternal deaths and perinatal deaths (stillbirth and neonatal deaths) in the world. The country accounts for up to 14% of global maternal deaths and is second only to India in the number of women who die during childbirth. Although all parts of the country are worsened by these staggering statistics, several lines of evidence show that most maternal, and perinatal deaths occur in the north-east and north-west geo-political zones where women have limited access to evidence-based maternal and neonatal health services. The proposed project intends to identify the demand and supply factors that prevent women from using PHCs for maternal and early new-born care in Nigeria, and to test innovative and community relevant interventions for improving women’s access to PHC services, and thus, ultimately, to prevent maternal and perinatal deaths.

**Methods:**

An open-labelled, randomized controlled trial will is carried out in two local government areas selected based on three criteria (i) maternal mortality rates (ii) PHC utilization rates and (iii) and geographic localization. The study will be conducted over 54-months in six communities, with PHCs in six communities of similar status serving as control sites. Surveys about quality of care and maternal health services utilization will be carried out at baseline, at midterm and at end of the project to test the effectiveness of the intervention, alongside conventional epidemiological measures of maternal and perinatal mortality. Ethical approval for the study has been granted (reference no. NHREC/01/01/2007). The findings will be published in compliance with reporting guidelines for randomized controlled trials.

**Discussion:**

The current Federal Government in Nigeria has identified PHC as its main strategy for increasing access to health in Nigeria. However, despite numerous efforts, there are persisting concerns that there is currently no scientific evidence on which to base the improvement of PHCs. The results of this study will identify barriers in the use of PHCs and will provide scientific evidence for effective and innovative interventions for improving PHCs that can be rolled out throughout the country.

**Trial registration:**

Clinical Trials.gov NCT02643953.

## Background

Current estimate of maternal mortality in Nigeria is 545 to 608 per 100,000 births [1, 2], which is among the highest in the world. Nigeria was listed in a Lancet publication as one of six countries accounting for 50% of global maternal deaths [2]. The estimated 40,000 maternal deaths in the country are the world’s second highest, and together with India, accounts for one-third of all maternal deaths worldwide. Nigeria’s stillbirth rate is 42 per 1000 and the second highest in the world, and is one of ten countries that account for up to 66% of stillbirths globally [2]. The World Health Organization (WHO) recommends increased access by women to evidence-based maternal and perinatal health care (antenatal, intrapartum and early postnatal care) as an essential intervention for improving maternal and perinatal outcomes in low-income countries [3–5]. This has been borne out by data showing that countries with the highest rates of skilled maternal health attendance also have the best indicators of maternal and perinatal health, even after controlling for other parameters of development [6–8].

Primary Health Care (PHC) is the recommended route for improving access to Skilled Birth Attendance (SBA) in low-resource settings. Several studies indicate that the major reasons that women fail to use maternal health services at PHCs are lack of money to pay for health services, difficulty with transportation, perceptions relating to the negative attitudes of health workers, and lack of permission from husbands and other family members [9–13]. A report of a Nigerian Presidential Committee titled “Accelerating the Attainment of MDG 5” in 2006 [14] identified high rate of poverty and the inability to pay for health services as major barriers experienced by women in accessing safe maternal care. To solve financial barriers, the Federal Ministry of Health in 2007 [14–19] recommended that State governments (the second tier of government in Nigeria) should implement policies on provision of free maternal and child health services. The Nigerian Primary Health Care Development Agency (NPHCDA) also established the Midwives Services Scheme (MSS) [16] for the purpose of staffing PHCs in under-served areas for the provision of maternal health care to address the supply challenges of lack of professionals in maternal health centres in many parts of the country. The results showed that by 2010, up to 18 out of the 36 Nigerian States were offering free maternal and child health services [17, 18]. In December 2012 [16], the NPHCDA reported that about 2500 midwives had been employed to work in PHCs in rural under-served communities across the country.

Despite the provision of free maternal care services, the experience in many states has been that health care utilization initially increased in some states, but this has not been sustained over time. Many health providers and women voiced concern that despite the free services, many health facilities lacked basic facilities, the health care personnel were poorly motivated, while many of the pre-existing challenges that led to poor utilization remained [19, 20]. The exception was Ondo State, which witnessed sustained and unparalleled transformation of maternal care, with the State achieving MDG-5 status, the only state to do so in the country. This was due to the institutionalisation of the *Abiye* program [21, 22]*,* which addressed both the demand and supply factors contributing to access barriers in a composite and multi-faceted manner. Under Abiye, the government provided free antenatal and delivery care, linked pregnant women to health providers through mobile phones and improved clinical service delivery at secondary health facilities. However, the fact that this intervention focussed on secondary care facilities (first referral level) only and was mainly located in urban areas without a research strategy to compare results with control sites diminished the policy relevance of this approach.

Evidence abounds to show that unskilled delivery dramatically increases the risk of maternal and perinatal death, as women who die during pregnancy, or who experience perinatal deaths, are often those not receiving antenatal, intrapartum and postnatal care, and those whose deliveries are attended by an unskilled birth attendant or who deliver at home [20–22]. National data from the Demographic and Health Survey [23] indicate that less than 65% of Nigerian women receive antenatal care during pregnancy, less than 33% are attended to by skilled birth attendants (SBA) at the time of delivery, and under 40% receive follow-up postnatal care.

In 2007, the Federal Ministry of Health developed and launched the Integrated Maternal, Newborn and Child Health (IMNCH) policy [24], to promote health equity for women and children. This policy emphasizes the provision of quality care for women right from the time of conception through pregnancy and the immediate postnatal period. Ensuring access to quality services for marginalised women was its focus, which the Ministry identified as the main operating framework of the document. To ensure that this policy can aid the most marginalized women in Nigeria, the Ministry adopted Primary Health Care as its main route of implementation. Nigeria’s health care system is based on a 3-tier system: Primary Health Care (PHC) as the entry point; Secondary Care (regional/general hospitals) as the first referral level; and Tertiary Care (teaching hospitals) as the second level referral. PHC is the most accessible and affordable form of care that serves most of the population, leaving substantially fewer women in need of Secondary and Tertiary care.

Nigeria presently has about 34,000 PHCs covering all the health wards (comprising population areas of 10,000 to 30,000 persons) and most rural and hard-to-reach communities. By contrast, the country has fewer Secondary Health Centres and Teaching Hospitals located mainly in large cities and urban areas. By focussing on the primary health system for implementing IMNCH, the idea is to reach the many poor and rural women at highest risk of maternal and perinatal mortality. Many such women can be effectively managed at PHCs, while the few who require more complex care (e.g. caesarean delivery or neonatal intensive care) would be transferred to higher level emergency care centres through the development of an effective referral system.

Despite a thoughtful system designed to reduce maternal and perinatal mortality in Nigeria, this goal has yet to be achieved among its most vulnerable and marginalized women. Many women still do not use PHCs for maternal and neonatal care, with a large proportion opting to use traditional forms of care or self-care. The problem has been compounded by PHCs that are inadequately staffed and equipped. Many PHCs are not aware of the updated guidelines for the management of pregnant women and neonates, and some PHCs are inadequately linked to secondary and tertiary care centres, for advice or patient transport. If the PHC system is to contribute effectively to reducing maternal and perinatal deaths in Nigeria, both the demand and supply sides of the system must be improved and strengthened. Women must be encouraged and empowered to use the PHC for maternity and early postnatal care. The PHCs must be improved and made more functional.

Despite the consensus of all stakeholders in Nigeria that the primary health care system is the key to reducing maternal and perinatal mortality, steps have yet to be taken to consolidate and revamp the system. In 2010, the Nigerian Primary Health Care Development Agency (NPHCDA) launched the Midwifery Services Scheme (MSS) to ensure the deployment of midwives to poorly staffed PHCs throughout the country. However, to date, the MSS has only attained modest success, while the questions regarding how to make the system work effectively and how to attract women to use the services still remain to be addressed. The National Assembly is currently debating a bill that will ensure that at least 1% (1 %) of Nigeria’s annual budget is allocated to improve the functioning of the PHC system. However, unless practical methods are in place for improving both the delivery of PHCs and the access of women to PHCs, greater funding alone will not achieve the desired results. The proposed project intends to identify the demand and supply factors that prevent women from using PHCs for maternal and early new-born care in Nigeria, and to test innovative and community relevant interventions for improving women’s access to PHC services, and thus, ultimately, to prevent maternal and perinatal deaths.

## Methods

### Theoretical framework

Three conceptual frameworks are considered in this study. The first is the three-delay model, earlier proposed by Thaddeus and Maine [19]. In brief, the model describes the interlinking nature of the different factors that prevent women from accessing evidence-based maternal and perinatal health care. The model identifies the different barriers that women face in achieving the care needed to prevent maternal and neonatal complications. This model identifies Type 1 Delay is the failure of a woman to seek help when she experiences pregnancy complications, while Type 2 Delay is delay due to difficulties with transportation to a health facility. By contrast, Type 3 Delay is the delay experienced when the woman arrives in the health facility.

The decision to use maternal health services is largely an individual choice, while the utilization of health services remains a complex and multi-facetted behavioral phenomenon. Thus, our second conceptual framework for this study is based on the *health seeking behavior model* developed by Anderson and Newman [25]. This model proposed that the use of health care services is a function of three sets of individual characteristics – predisposing characteristics, enabling characteristics and need characteristics. The model posits that the use of maternal health services is influenced by predisposing factors such as maternal age, education, household size, number of previous pregnancies and health related attributes. With respect to enabling characteristics, the framework proposes that access to health services and health personnel is an important determinant of maternal health care utilization. This implies that women’s ability to use maternal health facilities will depend on the availability of such facilities and their possession of the means to access the facilities. An important component of this framework – the need component – suggests that the use of maternal health services can be influenced by a woman’s perception of the relative importance of modern health care services versus traditional methods of care. Added to this is a woman’s perception and understanding of pregnancy complications and her desire to deliver safely and attain a healthy newborn baby.

In this proposed project, we will use a simplified definition of access to health services, as proposed by Peters et al. [26] who defined it as “the timely use of service according to need”. Therefore, a third conceptual framework that exemplifies this study is the four dimensions of access proposed by O’Donnell [27]. This includes availability, geographical accessibility, affordability and acceptability, while he further proposed that these could stem from both demand and supply sides. *Demand side* factors are those that relate to the ability to use maternal health services at individual, household or community levels. *Supply side* factors relate to the health care system that hinder service uptake by individuals, households and communities. In this regard, some demand side factors include costs of services (such as indirect costs related to transportation), information on health care services/providers, education, household expectations, community and cultural preferences.

We hypothesize that if we can reduce Type 1, 2 and 3 Delays, then we can reduce maternal and perinatal mortality and morbidity, especially if demand and supply side factors are addressed concurrently. Thus, this project aims to find out why delays occur in seeking maternal health care in PHCs, and then to test interventions to reduce all types of delays. We will also ensure that the tested interventions focus on most at-risk poor women in rural, urban and peri urban areas that use PHCs for antenatal, intrapartum and early postnatal care.

We will do so by generating new evidence, and complimenting those findings with the existing evidence about improving maternal health care delivery in low and middle-income countries. The project will be designed to maximize community participation and ownership. By having community members lead the intervention design and implementation, there is a better chance of improving women’s use of PHCs for maternal care in differing regions of Nigeria.

### Study design and setting

A community-based, multi-site, and multi-disciplinary cluster randomized trial using a mixed methods approach will be used. The project will be done in three phases: A formative phase (Phase 1) and an intervention phase (Phase 2), followed by evaluation of the intervention and policy transformation of the findings (Phase 3). The study will be conducted over 54-months in selected intervention communities, with selected communities of similar status serving as control sites. Surveys about maternal health services utilization will be carried out at baseline, at midterm and at end of the project to test the effectiveness of the intervention, alongside conventional epidemiological measures of maternal and perinatal mortality, each also collected in the three terms (Table 1).

**Table 1:**
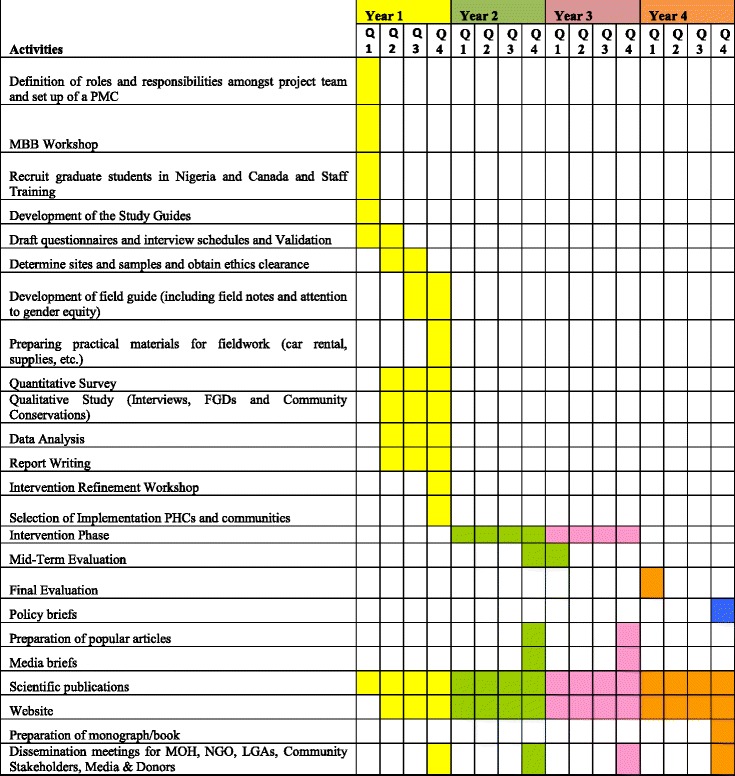
Project GANTT chart

### Research objectives

This project aims to reduce maternal and perinatal mortality in Nigeria by strengthening the availability and use of maternal primary health care services by vulnerable women.

The specific objectives of the project are as follows:

1. To identify the demand and supply factors responsible for use and non-use of primary health care for maternal health in selected communities in Edo State, Nigeria;
2. Based on Objective 1, to derive and implement a set of multi-faceted community-led interventions to increase women’s access to antenatal, intrapartum and postnatal care services in primary health centres in six (6) randomly selected communities in six geo-political zones of the country;
3. To evaluate the effectiveness of the implemented multi-faceted interventions by comparing both indicators of access to services, as well as maternal and foetal/new-born health outcomes, between the intervention-receiving communities vs. the control communities not receiving the intervention;
4. To use the study findings to modify Nigerian policy and programmes aimed at increasing women’s access to evidence-based maternal and neonatal services, especially within the country’s rural areas.

### Key research questions

The relevant questions that would be addressed by the study include the following:

1. What demand factors (acceptability, geographical accessibility, affordability etc.) account for the low utilisation rates of evidence-based prenatal and intrapartum care services within PHCs located in rural communities within Edo State, Nigeria?
2. What supply factors (healthcare providers, availability of medical equipment, essential drugs and infrastructure etc.) account for the low utilisation rates of evidence-based prenatal and intrapartum care services within PHCs located within rural communities in Edo State, Nigeria?
3. How do these demand and supply factors interact with each other?
4. What are women’s preferences for specific maternal health service delivery?
5. What are communities’ perceptions about maternal health and illness, including the possibility of death in pregnancy, and how do these beliefs and perceptions impact the use of evidence-based maternal health services by community constituents?
6. How do we develop community understanding and acceptance of the need to build women’s agency and capacity in accessing PHCs for maternal health?
7. What support can indigenous communities provide for women seeking maternal health services during pregnancy?
8. What types of interventions are effective in increasing the use of evidence-based prenatal and intrapartum care services in Edo State, Nigeria?
9. What is the best model for communicating the study results to policymakers?
10. What are the best ways to ensure that policy makers will use the study results and devote resources for improving MNCH indicators?

### Study area

Nigeria currently has a population of over 180 million, making it the sixth largest in the world after China, India, USA, Indonesia and Brazil. Nigeria is made up of 36 States and a Federal Capital Territory and has 774 Local Government Areas (LGAs). It is grouped into six geo-political zones/regions: North West, North East, North Central, South East, South West and South- South. The project is being conducted in two LGAs in Edo State, which we hope to scale up to other health care centres in the State and the country at large. Edo State is one of the 36 States of Nigeria with the population of over 3 million and total land area is 19,794sq.km. The study is being conducted in Esan South East and Etsako East LGAs in Edo State, South-South region of Nigeria. Both LGAs are located in the rural and riverine areas of the state, adjacent to River Niger, with Estako East in the northern part of the Edo State part of the river, while Esan South East is in the southern part. Administratively, each LGA comprises of 10 political wards and there are several communities in each ward (total of 100 communities from Esan South East and 42 from Etsako East). The two LGAs have a total population of 313,717persons, with Esan South East accounting for 167,721 and Etsako East LGA accounting for 145,996.

### Study phases

#### Phase 1: Formative research

##### Quantitative method

The quantitative instruments (individual woman, exit interview and site inventory assessment questionnaires) will be entered into a computer-assisted personal interviewing (CAPI) program. This method will explore the supply factors in the use or non-use of maternal health care services. A probabilistic sample will be carried out in the 20 communities to be selected out of a total of 142 communities in both LGAs, and will have two phases. In the first phase, 10 communities (5 PHC owned and 5 non-PHC owned communities) in each of the two LGAs will be chosen. All ever-married women aged 15 to 45 in each household to be visited who have an under-5 children will be eligible for interview to determine whether they have given birth within the period, and if so whether they have used the services of the PHC. This will continue until the required sample size of about 1450 women is achieved taking at least 72 women from each community. In addition, exit interview will be conducted in up to 10 selected health facilities with clients who would have received antenatal care, delivery or post natal care, to assess patients’ satisfaction through quality of care, patients’ time of travel or distance to facilities, and the cost of services at the PHCs.

In the second phase, PHCs/facilities assessment will be done through a checklist developed using the National Primary Health Care Development Agency standard. This will be used to assess the quality of infrastructural facilities, availability of clinical services through medical equipment and essential drugs as well as the availability of health care providers. All quantitative instruments will be designed and pre-tested to ensure that they are consistent to sufficiently answer related research questions.

##### Qualitative method

We will use participatory aspects of the qualitative data collection to engage these various stakeholders as follows:

**Key Informants Interviews:** We will conduct interviews with the communities and facilities stakeholders to determine their views on PHCs and how to improve its use by women seeking pregnancy care. Those views will be incorporated in the design of the project implementation.

**Focus Groups Discussion:** We will also conduct focus groups discussion (FGDs) with various categories of women to elicit their views on PHCs and pregnancy care, and feedback such views into the planning and design of the intervention. In particular, we will seek ways during the FGDs to determine how to include women’s perspectives through all phases of the implementation process.

**Community Conversations:** We would hold initial community conversations (CC) with community leaders in the identified project communities. During CC, we will bring together the decision-makers and leaders in each community to a village hall meeting to discuss the project ideas and intentions. We will introduce the project objectives and request them to identify ways the communities would help to solve the problems. The idea behind community conversation is to ensure that the communities identify the problems themselves and proffer solutions that they themselves are comfortable with. This way, not only will the communities participate fully in the project activities, they would also owe the project and sustain its implementation over time.

**High Level Advocacy**: We will pay advocacy visits to policymakers/decision-makers identified above to explain the project objectives and activities and to ensure that they provide resources (both human and financial) to undertake the improvements of the project PHCs. We hope to conduct high-level advocacy to enable decision-makers and policy-makers to prioritise the improvement of PHCs.

#### Implementation tools

The formative study (Phase 1) will utilize Equitable Impact Sensitive Tool (EQUIST) and Knowledge Translation (KT) strategy. An Intensive training on EQUIST, a tool for high-impact intervention and budgeting will be conducted to teach team members on the strategies for knowledge translation on the project using EQUIST as a guiding model to address health systems bottlenecks hampering the delivery of maternal care services, potential improvement and cost implication needed for the expected results. EQUIST as a planning tool to improve maternal and child health is helpful to identify how disadvantaged rural women can be targeted to utilize Primary Health Centres (PHCs) for maternal and child health care. The analysis will also enable the identification of why women are disadvantaged, especially highlighting the gender and social equity dimensions. It will also include an analysis of how the combination of evidence-based high impact interventions and health system strengthening strategies can produce the best results for the project. Other skills to be taught at the training will equip project team members on productive knowledge translation strategies including inter-sectorial collaboration in policy making and implementation, managing political interference in policy making and implementation, policy formulation and how the legislation process can be leveraged to support and sustain the project. The Knowledge Translation Platform (KTP) will utilize production of policy briefs on maternal health, advocacy visits to key stakeholders and decision makers in the LGAs, Edo State Ministry of Health, Federal Ministry of Health, the health practitioners fora, print and electronic media, the local NGOs, the intervention communities’ partners, research publication, and West African Health Organization (WAHO). This list will be complemented if needed and according to the circumstances on the field.

#### Phase 2: Design and implementation of the intervention

##### Intervention design workshop

Following Phase 1, we will organize an intervention workshop to design the intervention using Phase 1 research findings. The intervention workshop will also serve to disseminate Phase 1 research findings so as to build support for the project among critical stakeholders.

##### Intervention design

The intervention will consist of 1) strategic approaches such as information provided to pregnant women to encourage them to attend primary health care and use family planning, antenatal, delivery and postnatal services; 2) targeted community health education and advocacy activities; 4) community maternal audit/accountability activities, with community-led activities aimed at promoting utilization of services; 4) outreach services by PHCs; 5) PHC strengthening including training of health providers on treatment protocols and referrals; 6) enumeration of traditional birth attendants and training/motivating them to refer maternal and child health cares; and 7) training and kitting of community health rangers who will be trained to follow up women at home to ensure that they do not default but that they continue to use PHC services until delivery.

#### Phase 3: Monitoring and evaluation of the intervention

WHARC has an existing Monitoring and Evaluation Department that would be used to monitor and evaluate the project. In brief, both process and outcome indicators will be used to monitor the achievements of the specific objectives of the project. A logic framework will be designed that will capture each research objective, the expected outcomes, the indicators for measurement, means of verification and the identification of the specific reporting project official. Both quantitative as well as qualitative measures will be used to capture the results of the project. Through triangulation of the qualitative and quantitative results, a more comprehensive assessment of the results of the project and the intervention will be obtained.

The indicators for measuring the success of the project are being identified at three levels: indicators for the formative research phase (Phase 1), indicators for the intervention phase (Phase 2), and indicators of the translational research phase.

### Data source and sampling

A combination of secondary and primary data is required for the study. The sample size formula below is applied for the formative research survey as follows:

n_1_ = {[p_1_q_1_ + p_o_q_o_]) (Z_α/2_ + Z_β_)^2^}/ (p_1_-po)^2^.

p_0_ = utilization of PHC for maternal and perinatal in the control arm (assumed to be − 5 reduction in the prevalence in the experiment site).

p_1_ = utilization of PHC for maternal and perinatal care in the experimental arm.

*z*_*α*_ = Two-sided standard normal variate at 95% level of significance = 1.96.

*z*_*β*_ = Statistical power at 80% = 0.84;

n_1_ = n_2_.

n_1_ = no of study participants in the experimental group.

n^2^ = no of study participants in the control group.

We assume 50% since there is no literature from the geographical location of the study which reported the prevalence of utilization of PHCs for maternal and perinatal health care. Thus;

n^1^ = (0.50 × 1–0.50 + 0.45 × 1–0.45) (1.96/2 + 0.84)^2^/(0.50–0.45) 2.

(0.25 + 0.2475) (3.3124)/0.0025.

n^1^ = 659.

n^2^ = 659.

Total sample size = 1318.

10% adjustment for non-response = 132.

Total = 1450.

There will be 725 respondents in the experiment LGA and 725 in the control LGA.

### Statistical analysis plan

Baseline data will be summarized using descriptive statistics and percentages. No hypothesis testing will be performed on baseline data. However comparability of the LGAs will be evaluated from the perspective of clinically meaningful differences. The primary outcome SBA will be analyzed. The outcome data will be aggregated to the cluster level and analyzed with regression model to examine the determinants of skilled pregnancy care using PHCs. The Statistical Package for Social Science (SPSS) version 22.0 (SPSS Inc., Chicago, IL, USA) will be used to enter and analyse the data.

### Ethical clearance and registration

The ethical clearance approval needed for the project was obtained from the National Health Research Ethics Committee (NHREC) after the submission of the study protocol. The project ethical clearance certificate was approved on April 18, 2017, with NHREC Approval Number: NHREC/01/01/2007–18/04/2017. The protocol was also registered with ClinicalTrials.gov (Registration number is NCT02643953).

### Anticipated achievements

The primary achievements of this research will be: 1) the identification of the background determinants and reasons for poor use of health services for antenatal, intrapartum and postnatal care by Nigerian women; 2) the identification of potentially effective community and facility-based interventions to improve the supply and demand factors believed to be barriers to utilization of maternal health services by women; 3) the operationalization and testing of a minimal package for linking pregnant women to effective and evidence-based maternity health care in PHCs; and 4) the testing and determination of strategies for strengthening the health system to ensure effective linkage of primary health care to higher levels of care, and for improving the functioning of the primary health care overall.

Other anticipated achievements of this research include: 1) increased involvement and ownership of programs by participating communities through increased knowledge of evidence-based methods for promoting maternal health; 2) increased political commitment of the Local Government Councils in which the project is located, to providing resources and implementing strategies for reducing maternal and neonatal mortality; and 3) better training and motivation of health care personnel to provide primary maternity health care, especially within rural locations.

At the outset of the study, a monitoring and evaluation team will be formed that will develop and implement a detailed plan for the monitoring and evaluation and measurement of project successes. This will be done using both quantitative and qualitative methods, the examination of project and institutional records as well as the policy documents of participating Local and State Governments. Specifically, we will identify both the process and outcome indicators for tracking the achievements and successes of the project. Some of the indicators to be used for measuring the project’s achievements include: 1) the evidence generated in Phase 1 about the social, cultural and economic determinants of the patterns of maternal health utilization; 2) the evidence developed from the package of effective interventions for increasing women’s access to primary health care services for maternal and neonatal care; 3) the improved quality of maternal and neonatal care services and better delivery mechanisms within the intervention-group primary health centres; 4) the better linkages of maternal PHC services to secondary and tertiary care through identification of appropriate and time-bound referral services; 5) the evidence of community participation, learning and ownership of the project activities; 6) increased political commitment by participating Local and State Governments, measured by increased budget provision for maternal and neonatal care at PHCs; and 7) evidence of increased PHC staff training and capacity building (including training of Masters and PhD students) on the project activities.

Increasing women’s access to evidence-based maternity care is currently one of the most urgent actions to effectively improve major indicators of maternal and child health in Nigeria. Thus, part of the project implementation is to ensure that the findings of the project will be used to scale up interventions to improve maternal and child health across Nigeria. Thus, the strategic objective of this study is to ensure that it not only results in greater access to primary maternal and neonatal health care, but that evidence-based guideline and procedures are used in these primary health facilities.

## Discussion

The proposed study has strengths and limitations. One of the strengths is its feasibility. But there are few potential risks associated with this research project. One is the possible failure of the political system to address the supply factors responsible for non-use of PHCs for pregnancy and childcare. While this project would be able to take care of staff training, the re-building of iinfrastructure, staff employment, deployment and remuneration would have to be handled by responsible government departments and agencies. Without this, we would be not be able to achieve the milestones on improvement of supply factors that lead to poor demand of services. Our fear is that policymakers may be unwilling or unable to improve the supply of services. But we believe that intense engagement of policymakers through high-level advocacy, public health engagements and strategic communication of the project will help to hold government officials accountable to dealing with the problem. This is more plausible due to the recent prioritization of PHC by the national government and the passage into law of the national health bill that provides a better funding strategy for PHCs. Specifically, the intense advocacy and strategic communication activities that will be carried out as part of the project implementation at all levels will help to deepen the understanding of the project by government agencies, and therefore, mitigate the possible effects of this risk.

Additionally, we will work with officials at the Family Health Department of the Federal Ministry of Health, Abuja, to mitigate this risk. As the department that oversees maternal and child health activities throughout the country, our engaging them will ensure the full participation of the National Primary Health Care Development Agency (NPHCDA), the Edo State Government and Ministry of Health and the Local Government Councils in the project activities.

Another risk is the possibility that the targeted communities might not agree to participate in the project. However, our proposed methods for identifying and entering the communities would help to mitigate this risk. First, we would ensure that we contact the community leaders of the identified wards, and explain the purpose, methods and mission of the project. Only communities and wards whose leaders accept to participate in the fully explained project will be included in the project. Second, we will identify women groups and leaders in the project communities and explain the project to them. They will be encouraged .to then explain the project to the women who will be enrolled in the studies. Third, we will identify community contacts who will function as the community liaison group between the project team and each local community. The public health education activities to be carried out on the project will also help to deepen understanding of the project by stakeholders in each community.
